# Difficulty of diagnostic accuracy of periprosthetic joint infection: a retrospective analysis of revision surgery of total hip arthroplasty and total knee arthroplasty in a tertiary hospital

**DOI:** 10.1186/s12891-024-08071-z

**Published:** 2024-12-12

**Authors:** Alexander Herbert Andres, Juliette-Afi Chaold-Lösing, Hendrik Bulok, Roland Ernst Willburger

**Affiliations:** 1 Department of Anaesthesiology, Intensive Care and Emergency Medicine, Martin-Luther-Krankenhaus, Katholisches Klinikum Bochum, Voedestrasse 79, 44866 Bochum, Germany; 2 Department of Orthopaedic Surgery, Katholisches Klinikum Bochum, Ruhr-University Bochum, Gudrunstrasse 56, 44791 Bochum, Germany

**Keywords:** Total hip arthroplasty, Total knee arthroplasty, PJI, Periprosthetic joint infection, Mechanical loosening, Diagnostic criteria, Comorbidity, Joint arthroplasty, One-stage revision, Two-stage revision, Resection arthroplasty, Predictors, Risk factors, Assessment

## Abstract

**Background:**

Diagnostic accuracy of periprosthetic joint infection still remains controversial and an unsolved problem with respect to clinical signs and laboratory measures. Influencing factors of diagnosis like age, sex, abnormal physical findings and comorbidities are published with different results. The aim of our study was to find factors strengthening the diagnosis.

**Methods:**

We therefore retrospectively investigated all revision surgeries of total knee arthroplasty and total hip arthroplasty in the years 2019 and 2020 in a tertiary hospital with special regard to diagnostic accuracy of periprosthetic joint infection and switch in diagnosis to aseptic mechanical loosening or vice versa. All patients were divided into 4 non-hierarchical groups: suspected and discharge diagnosis periprosthetic joint infection (P1), suspected and discharge diagnosis mechanical loosening (P2), suspected diagnosis mechanical loosening and discharge diagnosis periprosthetic joint infection (P3), suspected diagnosis PJI and discharge diagnosis mechanical loosening (P4).

**Results:**

In the years 2019–2020, 106 patients underwent revision surgery of total knee arthoplasty and total hip arthroplasty. 33 patients showed periprosthetic joint infection (31.1%) according to Infectious Diseases Society (IDSA) criteria, 73 patients showed mechanical loosening (68.9%). Of the periprosthetic joint infection -patients, 15 were men (46%) and 18 were women (54%). The patients with mechanical loosening were 27 men (37%) and 46 women (63%). In group P1 (25 patients), 22 could be classified according to the histopathological classification Krenn and Morawietz grade 2 and grade 3, 2 patients to grade 4 and one patient could not be classified. In group P3 (8 patients) all could be classified according to the classification Krenn and Morawietz grade 2 and 3. Groups P1 – P4 were correlated with categorial basic data: All Groups P1 – P4 showed a statistical correlation towards American Society of Anesthesiologists (ASA) categorization 3–4 versus ASA 2 (*p* = 0.01). In the pairwise comparison in the exact Fisher´s exact test P1 and P2 showed a statistical correlation towards ASA categorization 3–4 versus ASA 2 (*p* = 0.01). Charlson Comorbidity Index (CCI) categories 5–7 versus 0,1–2 and 3–4 showed a statistical correlation towards groups P1-P4 (*p* = 0.007) and in the pairwise comparison in the exact Fisher´s exact test a discrimination of P1 and P2 (*p* = 0.001) and P1 and P3 (*p* = 0.007). The preoperative corticoid therapy showed a statistical correlation to groups P1-P4 (*p* = 0.05) and in the pairwise comparison in the exact Fisher test a discrimination of P1 and P4 (*p* = 0.02).

**Conclusion:**

We therefore conclude that diagnosis of periprosthetic joint infection still remains difficult. Switches in diagnosis during hospital stay from periprosthetic joint infection to aseptic mechanical loosening and vice versa are not unusual and the role of different diagnostic tools needs further investigation. Patients categorized according to ASA and CCI as severely ill might be more likely to be diagnosed correctly with periprosthetic joint infection.

**Supplementary Information:**

The online version contains supplementary material available at 10.1186/s12891-024-08071-z.

## Introduction

Periprosthetic joint infection (PJI) occurs in 1–2% of primary and in 4% to10% of revision arthroplasties [1–5]; a recent register analysis showed an incidence of 0,97% in THA (total hip arthroplasty) and 1,03% in TKA (total knee arthroplasty) [6]. Due to lifestyle changes and higher life expectancy, an increasingly elderly population and higher expectations for mobility in older age, the number of implanted prosthetic joints continues to rise [2, 7]. With a steadily increasing number of implantations, the number of PJI cases also rises. Development of detection methods for microbial biofilms helps to recognize even chronic infections that would previously have been missed. Management of PJI requires complex treatment strategies including surgical revision and long-term antimicrobial treatment (overview in [8]). Diagnostic accuracy of PJI with identification of the infecting micro-organism and its antimicrobial susceptibility is important for choosing the appropriate treatment strategy to eliminate the infection. When not diagnosed correctly or undertreated, PJI can lead to a persisting infection and multiple surgical revisions causing poor function or disability [3]. PJI definitions have set a clear focus on the need for accurate diagnosis and reference standards for clinical and diagnostic purposes. However, no uniform definition has prevailed. This can be due to many factors, above all use of expensive tests and disagreement over the accuracy of some of the included tests [9]. Early and accurate diagnosis is essential to effective management of PJI. A diagnosis within the first 2 weeks allows rapid treatment before a resistant biofilm is formed [10]. However, chronic infections can be “silent” or culturally negative by conventional tests. Many immunocompromised patients do not present with typical symptoms such as fever or increased neutrophil counts, so their symptoms may be mistaken for aseptic loosening, instability, or deformity [11]. Therefore, some infections are undetected at the time of surgery, leading to deterioration of periprosthetic tissue and patient condition [12]. Several orthopaedic and infection associations have published guidelines and expert opinions, starting with the Musculoskeletal Infection Society (MSIS) in 2011 [13], modified in 2013 [14]. Also published in 2013 by the Infectious Diseases Society (IDSA) [15], expanded in the following years [16–20]. No single diagnostic definition is acknowledged as a gold standard and up to now there is no precise definition for diagnosis of PJI and diagnostic accuracy remains a matter of debate. Influencing factors of diagnosis like age, sex, abnormal physical findings and comorbidities are published with different results [21, 22]. A recently published register-based study from the German Arthroplasty Register showed low unicondylar knee replacement implantation volume, comorbidities (elevated Elixhauser score), male sex, and implantation of constrained TKA are risk factors for septic revision after knee arthroplasty implantation [23]. Moreover, the study of synovial fluid biomarkers has increasingly come into focus in last years [24]. Further current research and development on technical topics enrich the debate and may show additional new areas for future research [25–27]. Hence, the amelioration of diagnostic accuracy and influencing factors is endorsed. With respect to the implementation of good healthcare practices in orthopedics, nowadays there are worldwide foundations like the PRO-IMPLANT-FOUNDATION [28] with workshops, online seminars, infection consulting and pocket guidelines, trying to integrate these diagnostic workflows in orthopedic healthcare facilities.

We therefore retrospectively investigated all revision surgeries of total knee arthroplasty and total hip arthroplasty in the years 2019 and 2020 at the Martin-Luther Hospital in Bochum, Germany with special regard to influencing factors of diagnostic accuracy of periprosthetic joint infection and switch in diagnosis to aseptic mechanical loosening or vice versa. Moreover, we aimed to compare the PJI and loosening groups to identify patient background factors that could potentially enhance the diagnostic accuracy of PJI.

## Methods

### Patients

We retrospectively investigated all revision surgeries of total knee arthoplasty and total hip arthroplasty in the years 2019 and 2020 in a tertiary hospital with special regard to diagnostic accuracy and switch in diagnosis to aseptic mechanical loosening or vice versa in a tertiary hospital.

### Inclusion criteria

Inclusion criteria were all orthopedic revison surgery with aseptic mechanical loosening or suspected or proved infection of the joint. Based on history and clinical examination, aseptic failure was defined as unexplained pain or a mechanical problem (loosening or wear revealed by X-ray imaging and clinical judgment) or both. Infection was suspected in presence of a communicating sinus, or an acutely unwell patient with fever and a swollen joint compatible with a haematogenous infection.

Inclusion in PJI was based on the diagnosis of PJI according to IDSA [15], since this could be defined with simple clinical means and histopathology. Definition of PJI: The presence of a sinus tract that communicates with the prosthesis is definitive evidence of PJI; the presence of acute inflammation as seen on histopathologic examination of periprosthetic tissue at the time of surgical debridement or prosthesis removal as defined by the attending pathologist is highly suggestive evidence of PJI; the presence of purulence without another known etiology surrounding the prosthesis is definitive evidence of PJI; two or more intraoperative cultures or combination of preoperative aspiration and intraoperative cultures that yield the same organism (indistinguishable based on common laboratory tests including genus and species identification or common antibiogram) may be considered definitive evidence of PJI [15].

Aseptic failure was suspected by the presence of pain during activities and radiological abnormalities or component migration or both. In TKA, mal-positioning and instability was judged clinically and supported by radiological examinations. In THA radiological examination was assessed for implant migration and radiological conspicuous features.

### Exclusion criteria

Exclusion criteria were all primary operations of TKA (total knee arthoplasty) and THA (total hip arthroplasty).

Patient´s characteristics are shown in Table 1.

**Table 1:** Patient´s characteristics

	All Patients	Patients with Periprosthetic Joint Infection (PJI)	Patients with Mechanical Loosening
Number of Patients	106	33 (31,1%)	73 (68,9%)
Mean Age, Years	63,9	62,6	64,9
Sex, number of males (%)	43 (40,6%)	15 (45,5%)	27 (36,9%)

A local document with guidelines and instructions was followed if there was a suspicion of PJI. A pragmatic evaluation with pre-operative examination was chosen in order to follow the existing practice in the department. In all cases the surgeon obtained the history and examined the patient. Ordering X-ray examination and routine inflammatory markers (leukocyte count in peripheral blood and plasma C-reactive protein) were standardized laboratory-confirmed.

Joint aspiration was encouraged as a primary diagnostic procedure but not always performed.

### Sample size

Diagnostic accuracy of periprosthetic joint infection still remains controversial and an unsolved problem even in 2024; we therefore reviewed in 2022 all patients operated on revision surgery of total knee arthoplasty and total hip arthroplasty within 2 years (2019–2020) and detected 106 patients.

The patients were divided into 4 groups: suspected and discharge diagnosis PJI (P1), suspected and discharge diagnosis mechanical loosening (P2), suspected diagnosis mechanical loosening and discharge diagnosis PJI (P3), suspected diagnosis PJI and discharge diagnosis mechanical loosening (P4).

All groups (P1 to P4) were correlated with the following categorial parameters (table Appendix 1). Since disease severity can have an influence as seen in ASA-, CCI- and CFS-categorization, the groups were also correlated with a stratified ASA- categorization (ASA 2, ASA 3–4), the stratified CCI-categorization (CC1 1–2, CCI 3–4, CCI 5–7) and the stratified CFS-categorization (CFS 2–4, CFS 5–7). The frequency of the different categorial characteristics was given for each of the groups in relation to the groups P1-P4.

The 4 groups were not correlated with the following parameters:

- Chronic alcohol abuse, as here the anamnestic information as well as a preoperative quantification are to be rated as unreliable.
- The temperature measured preoperatively, since all temperatures measured were below 38 °C, which means that severe acute infections are excluded and the statistical discrimination is not meaningful.
- Presence of rheumatoid arthritis, since there were only 3 patients with this diagnosis, which means that a statistical statement is not possible with certainty.
- Presence of a preoperatively positive blood culture, since there were only 3 patients.
- The microbiological result of the puncture preoperatively, since only 3 patients had a positive result.

Distribution of suspected and discharge diagnosis PJI, suspected and discharge diagnosis mechanical loosening, suspected diagnosis mechanical loosening and discharge diagnosis PJI, suspected diagnosis PJI and discharge diagnosis mechanical loosening are shown in Table 2.

**Table 2:** Distribution of: suspected and discharge diagnosis PJI,* suspected and discharge diagnosis mechanical loosening*,* suspected diagnosis mechanical loosening and discharge diagnosis PJI*,* suspected diagnosis PJI and discharge diagnosis mechanical loosening*

		Number of patients (%)
P1	suspected and discharge diagnosis PJI	25 (23,6%)
P2	suspected and discharge diagnosis mechanical loosening	45 (42,5%)
P3	suspected diagnosis mechanical loosening and discharge diagnosis PJI	8 (7,5%)
P4	suspected diagnosis PJI and discharge diagnosis mechanical loosening	28 (26,4%)

### Revision surgery

Surgical revision followed the departments routines including a sampling of five periprosthetic synovial tissue biopsies taken with separate instruments. According to the protocol five samples of periprosthetic synovial tissue, periprosthetic bone tissue, and swabs from the implant surface were taken along with any exchanged prosthetic components. Joint fluid was aspirated with a needle once the joint was exposed, but prior to incision of the capsule. Five samples made it feasible to perform parallel testing by bacteriological culturing for 14 days (routine 6 days). Final culture reports from standard tissue biopsies were available after 7 days as usual. However, surgeons were notified about late positive. Cultures (i.e. after day 6) if they deviated from the standard cultures.

### Data sources and analysis

Baseline characteristics of patients, comorbidities, previous history of the affected joint and prior antibiotic treatment to revision surgery were obtained. Supplementary data were obtained from medical records. Blood biochemistry values were obtained from the laboratory information system (ORBIS): C-reactive protein (CRP) (normal range ≤ 5 mg/L); white blood cell (WBC) (normal range: count 4.9–9.5.0/µL).

The metric characteristics are described using the mean (MW), standard deviation (SD), median, and minimum and maximum (Min-Max). The Kruskal-Wallis test is used to test whether the distribution of the characteristic is the same in the four groups.

Fisher’s exact test tests whether membership in P1-P4 and the characteristics are independent. The test results enable a global statement to be made as to whether there is a significant dependency or not. If the global test shows a significant difference, then in a second step the four groups are compared in pairs.

The metric characteristics (Table Appendix1) are described using the mean (MW), standard deviation (SD), median, and minimum and maximum (Min-Max). Whether the distribution of the characteristic is the same in the four groups was tested with the Kruskal-Wallis test.

All statistical tests are two-sided at a significance level of 0.05. Stata/IC 16.1 for Unix was used for statistical analysis.

## Results

In the years 2019–2020, 106 patients with revision operations of total knee and hip arthroplasty were operated on.

The mean age was 63.9 years with a standard deviation of 12.5 years, a minimum of 26 years, a maximum of 88 years and a median of 65 years.

There were 43 male patients (40.6%) and 63 female patients (59.4%).

33 patients showed PJI (31.1%) according to IDSA definition)[15], 73 patients showed mechanical loosening (68.9%). Of the PJI patients, 15 were men (46%) and 18 were women (54%). The patients with mechanical loosening were 27 men (37%) and 46 women (63%).

All 33 patients in the PJI group met IDSA criteria as follows: 29 of these patients were classified according to Krenn and Morawietz grade 2 and 3, 2 patients had Morawietz grade 4, in 2 patients the result was lost.

In addition, there is one patient with the Morawietz 2 grading in the suspected and discharge diagnosis of mechanical loosening group and one patient with the Morawietz 3 grading in the suspected PJI group and the discharge diagnosis of mechanical loosening; these patients did not fullfill IDSA criteria.

The patients were divided into 4 groups, Table 2 shows the distribution.

Of the 25 patients from group P1, 22 were classified according to Krenn and Morawietz grade 2 and 3, 2 patients grade 4, one patients´ result was lost.

All 8 patients in group P3 were classified according to Krenn and Morawietz grade 2 and 3.

The number of patients classified according to IDSA is identical to those classified according to MSIS 2 [18].

In Table 3, the frequency of the different categorial characteristics was given for each of the groups, e.g. number and percentage of patients with ASA 2 and ASA 3–4, in relation to the groups P1-P4.

**Table 3:** Categorical characteristics in relation to the groups P1-P4

		P1	P2	P3	P4	*p*-Wert*
ASA	2	2 (8.0%)	17 (37.8%)	0 (0%)	6 (22.2%)	
	3	22 (88.0%)	28 (62.2%)	9 (100%)	19 (70.4%)	
	4	1 (4.0%)	0 (0%)	0 (0%)	2 (7.4%)	
ASA (kat.)	2	2 (8.0%)	17 (37.8%)	0 (0%)	6 (22.2%)	
	3–4	23 (92.0%)	28 (62.2%)	9 (100%)	21 (77.8%)	0.010
CCI	0	1 (4.0%)	7 (15.6%)	1 (11.1%)	4 (14.8%)	
	1	2 (8.0%)	12 (26.7%)	3 (33.3%)	3 (11.1%)	
	2	0 (0%)	8 (17.8%)	3 (33.3%)	7 (25.9%)	
	3	7 (28.0%)	7 (15.6%)	0 (0%)	4 (14.8%)	
	4	7 (28.0%)	3 (6.7%)	2 (22.2%)	5 (18.5%)	
	5	4 (16.0%)	5 (11.1%)	0 (0%)	3 (11.1%)	
	6	1 (4.0%)	1 (2.2%)	0 (0%)	1 (3.7%)	
	7	3 (12.0%)	2 (4.4%)	0 (0%)	0 (0%)	
CCI (kat.)	0	1 (4.0%)	7 (15.6%)	1 (11.1%)	4 (14.8%)	
	1–2	2 (8.0%)	20 (44.4%)	6 (66.7%)	10 (37.0%)	
	3–4	14 (56.0%)	10 (22.2%)	2 (22.2%)	9 (33.3%)	
	5–7	8 (32.0%)	8 (17.8%)	0 (0%)	4 (14.8%)	0.007
CFS	2	0 (0%)	1 (2.2%)	0 (0%)	1 (3.7%)	
	3	3 (12.0%)	17 (37.8%)	1 (11.1%)	7 (25.9%)	
	4	10 (40.0%)	16 (35.6%)	3 (33.3%)	8 (29.6%)	
	5	5 (20.0%)	5 (11.1%)	4 (44.4%)	6 (22.2%)	
	6	4 (16.0%)	6 (13.3%)	1 (11.1%)	2 (7.4%)	
	7	3 (12.0%)	0 (0%)	0 (0%)	3 (11.1%)	
CFS (kat.)	2–4	13 (52.0%)	34 (75.6%)	4 (44.4%)	16 (59.3%)	
	5–7	12 (48.0%)	11 (24.4%)	5 (55.6%)	11 (40.7%)	0.109
BMI	BMI 16	0 (0%)	0 (0%)	0 (0%)	1 (3.7%)	
	N	7 (28.0%)	15 (33.3%)	3 (33.3%)	8 (29.6%)	
	Ü	1 (4.0%)	14 (31.1%)	2 (22.2%)	8 (29.6%)	
	A°I	8 (32.0%)	10 (22.2%)	1 (11.1%)	7 (25.9%)	
	A°II	3 (12.0%)	5 (11.1%)	2 (22.2%)	2 (7.4%)	
	A°III	6 (24.0%)	1 (2.2%)	1 (11.1%)	1 (3.7%)	
	N	7 (28.0%)	15 (33.3%)	3 (33.3%)	8 (30.8%)	
	Ü+ A1 + A2 + A3	18 (72.0%)	30 (66.7%)	6 (66.7%)	18 (69.2%)	0.988
Smoking	nein	17 (68.0%)	39 (86.7%)	9 (100%)	22 (81.5%)	
	Yes	8 (32.0%)	6 (13.3%)	0 (0%)	5 (18.5%)	0.143
Diabetes	No	17 (68.0%)	34 (75.6%)	9 (100%)	21 (77.8%)	
	Yes	8 (32.0%)	11 (24.4%)	0 (0%)	6 (22.2%)	0.288
Cortisone	No	20 (80.0%)	42 (93.3%)	9 (100%)	27 (100%)	
	Yes	5 (20.0%)	3 (6.7%)	0 (0%)	0 (0%)	0.050
Renal failure	No	12 (48.0%)	30 (66.7%)	7 (77.8%)	20 (74.1%)	
	yes	13 (52.0%)	15 (33.3%)	2 (22.2%)	7 (25.9%)	0.205
Grading renal	I°	7 (53.8%)	9 (60.0%)	1 (50.0%)	6 (85.7%)	
Failure	II°	1 (7.7%)	2 (13.3%)	0 (0%)	1 (14.3%)	
	III°	4 (30.8%)	4 (26.7%)	1 (50.0%)	0 (0%)	
	IV°	1 (7.7%)	0 (0%)	0 (0%)	0 (0%)	0.677
Anticoagulation	no	11 (44.0%)	28 (62.2%)	6 (66.7%)	17 (63.0%)	
	yes	14 (56.0%)	17 (37.8%)	3 (33.3%)	10 (37.0%)	0.431
History of	no	19 (76.0%)	38 (84.4%)	8 (88.9%)	22 (81.5%)	
cancer	Current/heald	6 (24.0%)	7 (15.6%)	1 (11.1%)	5 (18.5%)	0.796

* exacte Fisher-Test

Fisher’s exact test tests whether membership in P1-P4 and the trait are independent, with a significance level of 0.05.

If the global test showed a significant difference, the four groups were hereinafter compared in pairs in a second step (Table 4).

**Table 4:** Pairwise comparison of the groups (exact Fisher-Test) with categorical characteristics

		vs. P1	vs. P2	vs. P3
ASA (kat.)	P2	0.010		
	P3	1.000	0.044	
	P4	0.252	0.201	0.303
CCI (kat.)	P2	0.001		
	P3	0.001	0.625	
	P4	0.022	0.794	0.519
Cortison	P2	0.124		
	P3	0.293	1.000	
	P4	0.020	0.287	

Categorizations (strata) are given for those characteristics for which the statistical significance was calculated.

ASA (categorization 2, 3–4): there is a significant relationship between group membership and ASA (*p* = 0.010 (global test)). A pairwise comparison of the four groups shows a significant difference between P1 and P2 (*p* = 0.010) and P3 and P2 (*p* = 0.044). In group P2, ASA 3–4 with a proportion of 62.2% is significantly less frequent than in group P1 (92.0%) and group P3 (100%). In the other pairwise comparisons, there was no significant difference in terms of distribution to ASA categories 2 and 3–4 (*p* > = 0.201).

Table 5 shows the detected organisms in the Groups P1-P4.

**Table 5:** Detected organisms in the groups P1-P4

	Organisms	Number of cases
P1	Staphylococcus aureus (MSSA), no MRSA	4
	Coagulase-negative Staphylococcus ssp.	5
	Proprionibacterium	2
	Actinomyces	1
P2	Staphylococcus aureus (MSSA), no MRSA	3
	Coagulase-negative Staphylococcus ssp.	10
	Proprionibacterium	2
	Actinomyces	1
P3	Coagulase-negative Staphylococcus ssp.	5
	Proprionibacterium	2
P4	Staphylococcus aureus (MSSA), no MRSA	4
	Coagulase-negative Staphylococcus ssp.	18
	Bacillus subtilis	1
	Micricoccus luteus	2
	Proprionibacterium	7
	Actinomyces	1

Coagulase-negative Staphylococcus ssp. Comprise Staphylococcus capitis, Staphylococcus lugdenensis, Staphylococcus epidermidis, Staphylococcus saccharolyticus, Staphylococcus warneri, Staphylococcus hominis, Staphylococcus pettenkofferi

Abbreviations : MSSA MRSA, methicillin-sensible Staphylococcus aureus, MRSA, methicillin-resistant Staphylococcus aureus

All patients received prophylactic antibiotics, directly before surgery. Thirteen of 106 Patients received antibiotic tratment prior to revision surgery; two patients of them showed signs of sepsis, one case with urosepsis and one case with septic signes by pneumonia, both in groupe P2.

Table 6 shows the administered therapeutic antibiotics prior revision surgery.

**Table 6:** Therapeutic antibiotics prior revision surgery

	Antimicrobial	Number of cases
P1	Levofloxacin	1
P2	Amoxicillin	3
	Ciprofloxacin	1
	Clindamycin	1
	Vancomycin	1
P3	Ampicillin	2
	Rifampicin	1
P4	Ampicillin	1
	Flucloxacillin	1

## Discussion

Reviewing all revision surgeries of total knee arthroplasty and total hip arthroplasty retrospectively in the years 2019 and 2020 in a tertiary hospital with special regard to diagnostic accuracy of PJI and switch in diagnosis to aseptic mechanical loosening or vice versa we found that diagnosis of periprosthetic joint infection still remains difficult. Switches in diagnosis during hospital stay from periprosthetic joint infection to aseptic mechanical loosening and vice versa are not unusual and the role of different diagnostic tools needs further investigation. Patients categorized according to ASA and CCI as severely ill are more likely to be diagnosed correctly with periprosthetic joint infection.

### Diagnosis

The diagnosis of periprosthetic joint infection (PJI) of knee and hip is still a major challenge and no test currently offers absolute certainty even today [19, 35, 36]. The current diagnostic algorithm of PJI of knee and hip is based on a combination of clinical symptoms, laboratory tests of blood and synovial fluid, microbiological detection of micro-organisms and histopathological evaluation.

### Development of diagnostic criteria

Several orthopaedic and infection associations have published guidelines and expert opinions: The MSIS published a first definition in 2011 [13]. The focus here was on a fistula communicating with the prosthesis or a pathogen in two different cultures of fluid or tissue. Alternatively, 4 of the 6 secondary criteria are used for definition. These are: (a) elevated serum erythrocyte sedimentation rate (ESR) and serum C-reactive protein (CRP) concentration, (b) elevated synovial leukocyte count, (c) elevated synovial neutrophil percentage (PMN%), (d) presence of purulence in the affected joint, (e) isolation of a microorganism in one culture of periprosthetic tissue or fluid, or (f) greater than five neutrophils per high-power field in five high-power fields observed in histologic analysis of periprosthetic tissue. The MSIS has always emphasized that there is a certain degree of imprecision and that PJI can also be present with fewer than 4 secondary criteria. Likewise, the multiple detection of low-pathogenic micro-organisms may not be associated with a periprosthetic joint infection, just like the single detection of highly pathogenic ones [13]. There are no specific cut-off limits for biomarkers. These criteria were modified in 2013 [14] and subjected to an international consensus review by the International Consensus on Musculoskeletal Infection (ICM). Hereby the leukocyte esterase was added and clear thresholds for the biomarkers were formulated. In 2013, the IDSA published diagnostic criteria for standardization with an international group of experts [15]. Here, too, an existing fistula or the multiple detection of pathogenic germs or the single detection of highly pathogenic micro-organisms are in the foreground; evidence of an acute inflammation in the histopathological examination was also included [15]. In the further course, the criteria of the MSIS were expanded to include weightings [16, 17] and were agreed upon in the „Proceedings of International Consensus on Orthopedic Infections“ [18]. This definition was validated in 200 aseptic patients and in 222 patients with periprosthetic joint infections [16]: The two main criteria, fistula or two positive microbiological cultures, remain. The secondary criteria were scored, a score of 6 or greater indicates PJI, a score between 2 and 5 makes the diagnosis probable and a score under 2 unlikely. The number our patients classified according to IDSA is identical to those classified according to MSIS 2 [18], whereby it must be noted that of the minor criteria only the CRP was determined. The ESR, the leukocyte esterase in the punctate, the alpha defensin, the granulocytes in the punctate and the CRP of the synovia were not determined. In 2021, the European Bone and Joint Infection Society (EBJIS), supported by the MSIS and the Study Group for Implant-Associated Infections (ESGIAI) of the European Society of Clinical Microbiology and Infectious Diseases (ESCMID), published an extended definition [19]. EBJIS considers a PJI as confirmed solely based on the following criteria: (i) a sinus tract communicating with the joint and/or (ii) more than 3000 white blood cells per microlitre synovial fluid or a percentage of polymorphonuclear leukocytes above 80% and/or (iii) positive histology with five or more neutrophils seen in at least five high-power fields [19]). Therefore PJI could be diagnosed without positive microbiological cultures, a fact that should be interpreted with caution [20].

A recent meta-analysis examined the usefulness of synovial biomarkers in the diagnosis of periprosthetic joint infections, such as alpha-defensin, CRP leukocyte esterase, interleukine-6, interleucine-1beta and interleukine-17. All biomarkers showed a good benefit here, but for all without statistical significance; the best result was shown for alpha defensin with a 748-fold higher odds-ratio of a positive result in patients with periprosthetic joint infections [37]. Gehrke and colleagues prospectively evaluated a new minute rapid lateral flow version alpha defensin test in 223 patients and compared it with the MSIS criteria as the gold standard; The sensitivity of the alpha defensin test was 92.1%, the specificity and the positive predictive value were both 100%, with the negative predictive value being 95.2% with an accuracy of 96.9% [38]. In another publication by Renz and colleagues, the alpha defensin test was compared with the criteria of MSIS, IDSA and EBJIS, showing a high specificity of > 95% with a limited sensitivity of 84% (versus MSIS), 67% (versus IDSA) and 54% (compared to EBJIS), whereupon the authors recommend using the test only for confirmation purposes [39]. Measuring inflammatory indicators (e.g. CRP and erythrocyte sedimentation rate) in combination with clinical symptoms is currently the most effective method for early detection of PJI after joint replacement surgery [40]. The study investigated 987 cases of complete joint revisions for PJI and 386 cases with aseptic revision. Age, gender, comorbidity, serum CRP values, serum leukocytes, microbiology of preoperatively taken aspirations and at least 2 intraoperative biopsies and presence or absence of a fistula were recorded. The mean CRP value in the PJI group was 50.2 mg/l (SD = 62.2), while the CRP value in the control group with aseptic revisions was 11.6 mg/l (SD = 25.3). There were no significant differences in CRP values between patients with and without fistula (*P* = 0.4423); the difference in CRP between high and low virulence micro-organisms was significant for both hip and knee (*P* < 0.0001). The optimal cut-off limit for CRP as a diagnostic marker for PJI, calculated using the Youden index, was 8.90 mg/l for the hip and 9.99 mg/l for the knee [40]. In a recent single − retrospective, observational cohort study with 77 consecutive cases in 4 years the authors found a statistically significant differences (*p* < 0.005) for neutrophil count, Lymphocyte count, haematocrit level, haemoglobin level,  CRP, the neutrophil lymphocyte ratio, platelet lymphocyte ratio, monocyte lymphocyte ratio, systemic inflammation index, systemic inflammation response index, and aggregate inflammation systemic index. The ROC curve analysis showed that the systemic inflammation index and the neutrophil lymphocyte ratio are the most accurate biomarkers [41].

As group P4 with suspected diagnosis PJI and discharge diagnosis mechanical loosening comprises a quarter of our study population all showed microbiological findings of primarily unclear and then clinically irrelevant significance. We would like to show, that finding of microbials is not necessarily linked to the diagnosis of PJI. It is of importance to note that there might be positive bacteriological findings without classifying the patients to a PJI. We consider them as commensals.

### Differential diagnosis

In a recently published review, 13 systematic meta-analyses were identified from 278 studies with 27,754 patients. There were 13 diagnostic tests with 7 categories, of which implant sonication (17.2), bacteriology (15.3), and differentiated cytological examination of the synovia (13.3) had the highest probability of a positive prediction. The highest negative predictive values were serum IL-6 markers (0.03) and differentiated synovial cytology (0.12) [36]. Koh and colleagues retrospectively reviewed the records of 303 patients who underwent two-stage knee revision at 17 facilities [21]. Data were also collected when the case did not meet MSIS criteria. Of the 303 patients, 198 met the diagnostic criteria proposed by MSIS. Of 105 patients who failed major MSIS criteria, 88% had two or three minor criteria, with 85% lacking preoperative knee puncture or histological work-up. The most common justification for the diagnosis of PJI was the presence of abnormal physical findings. Micro-organisms were only identified in 52% of all patients; the most common one was coagulase-negative Staphylococcus aureus. The authors conclude that the diagnosis of PJI was based on clinical suspicion in about one third of the cases. The study of Beretza and collegues investigated a total of 16 patients (11 women and five men) with diagnosed knee or hip implant loosening (mean implant survival of 102.1 months); the ultrasound procedure followed by PCR and sequencing improved bacterial identification in silent prosthetic joint infection and the lack of clinical signs of infection and negative preoperative and intraoperative cultures did not exclude.The presence of micro-organisms on the implants [11].

### Comorbidity all

There are several rating scales to decribe patiens: In short, the ASA-classification is a scale based on the doctors´ evaluation to describe a patients functional and body status before the operation [29]. The CCI categorizes and weights the severity of 19 different diseases and predicts the 30-day, 90-day and 1-year mortality as a continuous variable in patients with breast cancer [30] and in further evaluations in elective surgery [31]. In the original study from 1987, stratification into 4 groups already takes place: CCI 0, 1–2, 3–4 and CCI ≥ 5 [30]. The most important risk factors for periprosthetic joint infections were identified in a recent systematic review and meta-analysis. This analysis included 37 studies involving 2,470,827 patients. Older age was a protective factor (mean difference (MD) = − 1.18). Male gender, (odds-ratio, OR 1.34), coagulopathy (3.05), congestive heart failure (2.36), diabetes mellitus (1.80), obesity (1.61), systemic neoplasia (1.57), chronic lung disease (1.52) and hypertension (1.21) increased the risk of PJI. Behavioral risk factors such as chronic alcohol abuse (2.95), immunosuppressive therapy (2.81), steroid therapy (1.88) and nicotine abuse (1.82). Rheumatoid arthritis, post-traumatic native joint disease, high National Nosocomial Infections Surveillance (NNIS) system, surgical patient index score, prior joint surgery, and ASA status ≥ 3 were also significantly associated with a higher risk of PJI [22]. A similar setting was found in two smaller previous meta-analysis. The main factors associated with infection after total joint arthroplasty (TJA) in this PubMed, Embase and Cochrane search up to December 2015 were male gender (OR, 1⋅48; 95% CI, 1.19 to 1.85), age (SMD, − 0 × 10; 95% CI, − 0.17 to − 0.03), obesity (OR, 1 × 54; 95% CI, 1 × 25 to 1.90), alcohol abuse (OR, 1.88; 95% CI, 1.32 to 2.68), American Society of Anesthesiologists (ASA) scale > 2 (OR, 2.06; 95% CI, 1.77 to 2.39), operative time (SMD, 0.49; 95% CI, 0.19 to 0.78), drain use (OR, 0.36; 95% CI, 0.18 to 0.74), diabetes mellitus (OR, 1.58; 95% CI, 1.37 to 1.81), urinary tract infection (OR, 1.53; 95% CI, 1.09 to 2.16), and rheumatoid arthritis (OR, 1.57; 95% CI, 1.30 to 1.88; [42]. Sixty-six observational studies (23 prospective cohort and 43 retrospective cohort or case control) with data from 512,508 participants were included. Comparison of males to females and smokers to nonsmokers showed the pooled RRs for PJI to be 1.36 (1.18–1.57) and 1.83 (1.24–2.70), respectively. There was no significant correlation of PJI with age and high alcohol consumption. The RR values for BMI > 30 versus < 30 kg/m^2^, > 35 versus < 35 kg/m^2^, and > 40 versus < 40 kg/m^2^ were 1.60 (1.29–1.99); 1.53 (1.22–1.92); and 3.68 (2.25–6.01 [43]).

### Comorbidity – ASA and CCI

Various studies have shown a proportional relationship between comorbidity via ASA classification and the complication rate after elective orthopedic surgery such as knee and hip replacement. ASA-classification is a scale that relies on a physician’s assessment to describe a patient’s physical status before surgery. This classification was initially designed only as a description of the physical condition of the patients, taking into account their diseases [29]. Numerous studies subsequently showed that the ASA classification correlates well with the perioperative risk [44] and therefore it allows an estimation of complications and mortality perioperatively; since first description a division between ASA 1 and 2 and ASA ≥ 3 has been described.

In the study of Ondek and colleagues, comorbidity indices and demographics from National Surgical Quality Improvement Program in THA patients were evaluated for discriminative ability in predicting adverse outcomes using the area under the curve analysis from the receiver operating characteristic curves. Perioperative outcomes included any adverse events, severe adverse events, minor adverse events, extended hospital stay, and discharge to higher-level care. In total, 64,792 THA patients were identified and the most predictive comorbidity index was ASA, and demographic factor was age [45]. In a recent retrospective study with 1191 elective hip and knee prostheses 84 (7%) patients of the total cohort developed Superficial surgical site infection (SSSI). This infection progressed to a PJI in 24 (29%) of the patients. Factors with increased adjusted risk ratios (aRRs) for superficial surgical site infection (SSSI)s were knee surgery (1.7; 95% CI: 1.1 to 2.7), age ≥ 65 years (1.7; 95% CI: 1.1 to 2.8), BMI ≥ 30 (1.9; 95% CI: 1.0 to 3.4) and ASA classification ≥ 3 (1.7; 95% CI: 1.0 to 2.9). ASA classification ≥ 3 was the only factor showing a significant progression from SSSI to PJI (aRR = 3.3; 95% CI: 1.0 to 10.3 [46]), . In contrast to this, in a small retrospective study a total of 57 patients with 13 cases of acute PJI and 44 of chronic PJI, overweight patients (BMI > 25 kg/m^2^) represent a significantly larger proportion in both PJI groups (*p* < 0.05). Current smokers had a significantly increased risk for acute and chronic PJI (*p* < 0.05). In the acute PJI group 46.2% of patients had a postoperative urinary tract infection, but showed no correlation to ASA status [47].

The ACS NSQIP-database with 11.814 patients with 30-day readmission rates after elective primary TKA and THA revealed age (*P* = 0.002), male gender (*P* = 0.03), cancer history (*P* = 0.008), elevated blood urea nitrogen (BUN) (*P* = 0.002), a bleeding disorder (*P* < 0.001) and high ASA class (*P* < 0.001) as predictors of readmission in multivariate analysis [48]. The National Surgical Quality Improvement Program database identified all patients undergoing aseptic revison total hip arthroplasty between 2006 and 2013 in a multivariable regression model controlling for the significant perioperative variables, operative time, and ASA-classification. Higher CCI scores were significantly associated with mortality (odds ratio [OR] 1.89, 95% confidence interval [CI] 1.64 to 2.18, *P* < 0.001), major complications (OR 1.12, 95% CI 1.05 to 1.20, *P* = 0.001), minor complications (OR 1.53, 95% CI 1.39 to 1.69, *P* < 0.001), transfusions (OR 1.14, 95% CI 1.09 to 1.20, *P* < 0.001), and prolonged length of stay (OR 1.32, 95% CI 1.26 to 1.39, *P* < 0.001; [49]).

### Corticoid therapy

With respect to corticoid therapy the register-based cohort study using the Danish National Patient Registry, the DANBIO rheumatology register (rheumatoid arthritis-specific confounders and treatment episodes) and the Danish Hip and Knee Arthroplasty Registers showed in 3913 patients with RA with THA/TKA that patients with RA had a decreased 10-year risk of revision while the risk of death and that glucocorticoid exposure and increased disease activity were associated with an increased risk of death [50]. Rheumatoid arthritis, and long-term (> 1 year) corticosteroid use were also shown to increase the risk of surgical site infection after shoulder arthroplasty [51]. On the other hand a systematic review including 29 studies, categorized as using low- or high-dose corticosteroid with single or repeat dosing regimens, showed that single low-dose corticosteroid administration was not associated with an increased risk of infection (*p* = 0.4; CI = 0.00 to 0.00). Single high-dose corticosteroid was not associated with an increased infection risk (*p* = 0.3; CI = 0.00 to 0.01) nor did repeat low-dose regimens result in increased risk of infection (*p* = 0.8; CI = -0.02 to 0.02) [52].

### Histopathological investigations

Histopathology evaluation has a high specificity in predicting culture positive PJI and therefore confirming the presence of infection as shown in a systematic review by Tsaras [53]. On the other hand infections caused by low virulent organisms that fail to induce a neutrophilic reaction and pathologist expertise may contribute to the low sensitivity reported in some studies [54]. In a recently published trial, histopathological tissue analysis using the EBJIS criteria (cut-off of ≥ five PMN/HPF) showed a sensitivity of 93% and a specificity of 84%. Applying the IDSA and ICM criteria (≥ ten PMN/HPF) showed sensitivities of 94% and 90%, and specificities of 86% and 92%, respectively [55].

Whether in the two patients in the group suspected and discharge diagnosis PJI, who showed a Morawietz 4 graduation, this finding only represents a faulty sampling or the sensitivity is not 100%, cannot be finally clarified at the moment.

### Prevalence and outcomes of unexpected positive cultures

Most studies have been designed to detect standard infections with good sensitivity and specificity, and have used internal references for comparison. Diagnostic accuracy remains a matter of debate, even in case of the occurence of unexpected positive intraoperative culture (UPIC) in presumed aseptic revision arthroplasty surgery. Most of them used culture samples as gold standard, others histopatholgy and others the published guidelines with a mixture of them. For example, Atkins and colleagues used the in 1998 pathology diagnosis as the gold standard to which they compared the culture results [56]. In 1996, Tsukayama et al. first described a group of patients that underwent presumed aseptic revision arthroplasty surgery but had positive microbiological findings in the intraoperative tissue samples [57].

In our study the evaluation was based on the diagnosis of PJI according to the IDSA standard [15], since this could be defined with simple clinical means and histopathology. In recent reviews of the literature unexpected positive intraoperative culture in revision hip and knee arthroplasty has a prevalence of around 10%; there is no consensus in the literature on how to interpret them, above all when a single culture is positive [58, 59], whereas the incidence ranges between 5,9% [60] and 25% [61]. The occurence of unexpected positive intraoperative culture (UPIC) diagnosed via intraoperative culture without histopathologic examination is widely accepted [13, 59–65]. As well as the occurence of unexpected positive intraoperative culture (UPIC) diagnosed via intraoperative culture and histopathologic examination [66, 67]. In the literature, there is an inconsistency about the occurence of positive intraoperative culture and positive histopathologic examination at the same time: there are descriptions of a positive histopathologic examination without positive intraoperative cultures [11, 66] and reports of negative histopathological examination (Type Krenn and Morawietz 1 and 4) with relevant positive intraoperative cultures in all samples [68]. In patients with unexpected positive intraoperative cultures in presumed aseptic revision arthroplasty who were treated with antibiotics according to institutional criteria, the incidence of PJI after revision was higher in those who did not meet MSIS criteria (22%) than in those that met MSIS criteria (14%; *p* > 0.71 [37].

Taken together, up to now there is no precise definition for diagnosis of PJI and diagnostic accuracy remains a matter of debate, even in case of the occurence of unexpected positive intraoperative culture in presumed aseptic revision arthroplasty surgery. Moreover, the tool kit to diagnose with culture samples, histopatholgy or published guidelines or with a mixture of them remains a debatable point.

Severe other associated comorbidities comprise also concurrent infectious endocarditis in PJI-patients, which is a catastrophic and fatal complication and scenario. A recent case report of 18 joints in 16 patients showed data from five hospitals within an academic healthcare system in the northeastern United States over 20 years and documented high morbidity and mortality [69].

### Limitations

Our study has some limitations that should be stated. Firstly, these data are collected retrospectively. Secondly, our study comprises a small groupe with 106 patients with revision operations of TKA and THA at a single center. Additionally, the diagnostic algorithm applied in these years was not uniformly standardized. The sample size calculation was difficult, because there are no data about the amount of difference in the switched groups including confounding factors and comorbidities, therefore the statistical power is poor.

## Conclusions

Treating the underlying infection with success while preserving joint function, the treatment of PJI must include effective diagnosis and treatment based on algorithms and interdisciplinary collaboration all adapted to the patient. Up to now there is no precise definition for diagnosis of PJI and diagnostic accuracy remains a matter of debate.

We therefore conclude that diagnosis of PJI remains still difficult. Switches in diagnoses during hospital stay from PJI to aseptic mechanical loosening and vice versa are not unusual and the role of different tools like samples and histopathology is not still discussed fully. Our study suggests that severely ill patients categorized according to ASA and CCI with periprosthetic joint infection might be more likely to be diagnosed right.

A large area of future research and development remains open; new diagnostic methods with more accuracy, simplicity, and convenience are necessary fore widespread application and practicability. Pathogen-specific markers are currently tested for early and quick detection of PJI.

A grand field also remains in management of biofilm-related infections, including also active or passive implant coating or controlled local antibiotic application.

## Electronic supplementary material

Below is the link to the electronic supplementary material.

Supplementary Material 1

## Data Availability

All materials and data described in the manuscript, including all relevant raw data, are freely available to any scientist who wishes to use them for non-commercial purposes without breach of participant confidentiality and will be sent by the corresponding author upon request (alexander.andres@klinikum-bochum.de).

## Abbreviations

AF: Aseptic failure
ASA: American Society of Anesthesiologists, CCI: Charlson Comobidity Indes, CFS: Clinical Frailty scale
CI: Confidence interval
CRP: C-reactive protein
MD: Mean difference
MW: Mean
PJI: Prosthetic joint infection
SD: Standard deviation
THA: Total hip arthroplasty
TJA: Total joint arthroplasty
TKA: Total knee arthroplasty
OR: Odds-ratio
WBC: White blood cell count
