# Supplementary material for: Difficulty of diagnostic accuracy of periprosthetic joint infection: a retrospective analysis of revision surgery of total hip arthroplasty and total knee arthroplasty in a tertiary hospital
Source: BMC Musculoskelet Disord. 2024 Dec 12;25:1008. doi: 10.1186/s12891-024-08071-z (PMC11636231; doi:10.1186/s12891-024-08071-z)
Supplement: Supplementary file 1 — Supplementary Material 1 [file 12891_2024_8071_MOESM1_ESM.docx]

**Appendix-Tables of the manuscript**

**“Difficulty of diagnostic accuracy of periprosthetic joint infection: a retrospective analysis of revision surgery of Total Hip Arthroplasty and Total Knee Arthroplasty in a tertiary hospital”**

**by Alexander Herbert Andres, MD, MHBA, Juliette-Afi Chaold-Lösing, MD, Hendrik Bulok, MD and Roland Ernst Willburger, MD, Professor for Orthopaedic Surgery**

**Table Appendix1:**

***Categorial parameters were correlated with the groups P1 to P4,***

|  | ASA (American Society of Anesthesiologists) status (Anesthesiological classification to differentiate the severity of previous illnesses)  ASA 1: No previous illnesses  ASA 2: Pre-existing conditions that do not affect life  ASA 3: Pre-existing conditions affecting life  ASA 4: Life-threatening previous illnesses  ASA 5: moribund  Strata: ASA 2 and ASA 3-4  ASA-classification is a scale based on the doctors´ evaluation to describe a patients´ functional and body status before the operation [29], strata according to the original paper. |
| --- | --- |
|  | CCI (Charlson Comorbidity Index)  Classification to distinguish the severity of previous illnesses with regard to life expectancy, Strata 0,1-2 and 3-4  The CCI categorizes and weights the severity of 19 different diseases and predicts the 30-day, 90-day and 1-year mortality as a continuous variable in patients with breast cancer [30] and in further evaluations in elective surgery [31], strata according to the original paper. |
|  | CFS (Clinical Frailty Scale)  Classification to distinguish the severity of previous illnesses, especially mobility and frailty  Graduation 1-9, Strata: 1-4 and 5-8  The Clinical Frailty Scale (CFS) is a judgment-based assessment predicated on the cumulative deficit model that mixes items such as comorbidity, cognitive impairment, and disability and is validated [32].  1 Very Fit – People who are robust, active, energetic and motivated. These people commonly exercise regularly. They are among the fittest for their age.  2 Well – People who have no active disease symptoms but are less fit than category 1. Often, they exercise or are very active occasionally, e.g. seasonally.  3 Managing Well – People whose medical problems are well controlled, but are not regularly active beyond routine walking.  4 Vulnerable – While not dependent on others for daily help, often symptoms limit activities. A common complaint is being “slowed up”, and/or being tired during the day.  5 Mildly Frail – These people often have more evident slowing, and need help in high order IADLs (finances, transportation, heavy housework, medications). Typically, mild frailty progressively impairs shopping and walking outside alone, meal preparation and housework.  6 Moderately Frail – People need help with all outside activities and with keeping house. Inside, they often have problems with stairs and need help with bathing and might need minimal assistance (cuing, standby) with dressing.  7 Severely Frail – Completely dependent for personal care, from whatever cause (physical or cognitive). Even so, they seem stable and not at high risk of dying (within ~ 6 months).  8 Very Severely Frail – Completely dependent, approaching the end of life. Typically, they could not recover even from a minor illness.  9 Terminally Ill - Approaching the end of life. This category applies to people with a no life expectancy.  In 2005, Rockwood developed a 7-point Clinical Frailty Scale (CFS), based on a multidimensional approach including fitness, functional autonomy and comorbidities [32]. The scale then evolved to the 9 point-CFS, a visual scale classifying patients from fit (CFS 1–3), to vulnerable (CFS 4), to frail and more (CFS 5–9) [33]. |
|  | BMI (Body Mass Index),  Body Mass Index (BMI), kg/m^2^  Normal weight (18.5-24.9 kg/m^2^)  Overweight (25-29.9 kg/m^2^)  Obesity °I (30-34.9 kg/m^2^)  Obesity °II (35-39.9 kg/m^2^)  Obesity °III (>40 kg/m^2^),  Strata: normal weight and overweight versus Obesity I-III  The WHO defines overweight as a BMI greater than or equal to 25 kg/m^2^, while obesity is a BMI greater than or equal to 30 kg/m^2^ [34]. |
|  | Smoking status |
|  | Presence of diabetes mellitus therapy |
|  | Presence of cortisone therapy |
|  | Presence of chronic renal failure |
|  | Presence of preoperative anticoagulation |
|  | Preoperatively measured laboratory value CRP (C-reactive protein) |
|  | Presence of a history of tumor diseases, current or healed |
